# Youth and Tanning: A Dangerous Combination

**Published:** 2013-05-01

**Authors:** Pamela Hallquist Viale

As I write this from my home office in the Santa Barbara area, it is late March and the weather is a balmy 82 degrees with abundant sunshine and a promise for more of the same for the entire week. In fact, March weather in California is frequently sunny and warm, a fact that I took advantage of in my teens to begin my "starter" tan for the coming spring and summer seasons. My girlfriends and I would take bottles of baby oil and iodine and slather ourselves, basting in the early spring sunshine. I honestly do not remember being admonished by adults or health-care professionals to avoid the sun, although this was the late 1960s. In fact, my mother often joined me. And although I’ve yet to discover a skin cancer from those youthful skin indiscretions, I am always watchful, worried that my intense tanning from those early years will produce a malignant result.

Today, teens do not have to wait until the dawn of warm weather to begin their "starter" tans. In fact, they can tan all year long due to the many tanning salons that exist across the United States. A disturbing study recently published in the journal Pediatrics remarked on the fact that tanning facilities in Missouri frequently misstated the risks and dangers of tanning salons to consumers (Balaraman, Biesbroeck, Lickerman, Cornelius, & Jeffe, 2013). Even more alarming, younger children (as young as 10 or 12 years old) were often allowed to use the tanning facilities.

## Missouri Tanning Salons

The study authors conducted a statewide telephone survey of randomly selected tanning facilities in the state of Missouri, interviewing each salon twice to determine the reliability of the information obtained. Although 40% of the tanning facilities did mention the risk of skin cancer, 20% stated there was no risk if precautions were taken, and 31% didn’t mention anything on the risks of indoor tanning (Balaraman et al., 2013). A distressing 65% of salon operators reported that they would allow children as young as 10 or 12 to use their tanning devices, and although 77% required on-site parental control, only 47% requested on-site consent in the evening hours. And 17% claimed that an adult wasn’t necessary for children of this age group to use the indoor tanning facilities.

## Evidence of UV Radiation Exposure and Skin Cancer

As the amount of UV radiation exposure has increased among young adults, so has the incidence of skin cancer. A pivotal study published in 2002 suggested that the increased use of artificial tanning devices emitting UV radiation was contributing to the incidence of nonmelanoma skin cancers. In a population-based, case-control study of 603 basal cell carcinoma (BCC) patients and 293 squamous cell carcinoma (SCC) patients, the use of tanning devices was associated with odds ratios of 2.5 (95% confidence interval [CI] = 1.7–3.8) for SCC and 1.5 (95% CI = 1.1–2.1) for BCC (Karagas et al., 2002). Early age at first use was associated with a higher risk of BCC.

Recently, Zhang and colleagues (2012) demonstrated that there is evidence for a dose-response relationship between tanning bed use and risk of skin cancers, particularly BCC, with a stronger association in younger age exposures. The authors examined a population of 73,494 nurses for a period of 20 years, with 5,506 nurses diagnosed with BCC, 403 with SCC, and 349 with melanoma (Zhang et al., 2012). They determined a significantly higher risk of BCC with use of tanning beds during high school and college compared with no use during those years.

## Vulnerable Populations

The incidence of skin cancer, including melanoma, is rising; young children and teenagers are particularly vulnerable. These are the same individuals who often frequent tanning salons. An anonymous survey given to students in grades 9 through 12 (N = 368) revealed that more than 80% perceive movie stars as tan and that tan people are more attractive (Ashinoff, Levine, Steuer, & Sedwick, 2009). Many of the respondents were aware of the relationship between sun tanning and risk of skin cancer but stated they would continue to tan as an adult; the researchers felt that the perceived "invincibility" of teenagers and long time to development of actual skin cancer affected their beliefs. The authors concluded that national legislation to limit access of tanning salons to teenagers should be enacted based on their data. And although the US Food and Drug Administration requires a clearly visible warning sign on each tanning machine, a study demonstrated that one-third of observed tanning machines in New York City had no signage. The authors noted that 47 of the 85 salons visited had no warning signs whatsoever, and many of the existing signs in others studied were difficult to find (Brouse, Basch, & Neugut, 2011).

## Role of the Advanced Practitioner

I will admit it: As a youth, I thought I looked healthier with a tan. However, the data are convincing. All advanced practitioners working with this at-risk population of young adults should incorporate information about tanning and the risk of skin cancer into their patient assessment and education visits. Young patients with existing tans should be queried regarding their use of tanning beds; assessment of the knowledge and beliefs regarding tanning should be undertaken. It takes considerable time before the development of a skin cancer, but the risk of this cancer is increasing in this population.
